# Financing a greener future

**DOI:** 10.1038/s41467-023-36036-8

**Published:** 2023-02-07

## Abstract

Climate change is an expensive collective problem that will be felt unequally around the world and within communities. Sustainable and equitable financing is needed to address disparities and reduce the economic costs of loss and damage from climate change.

The impacts of climate change will not only have a great environmental cost, but also economic. More frequent exposure to climate extremes, including heatwaves, drought, flooding and storms, equate to not only a higher likelihood of infrastructural damage, but loss of life, livelihoods and production of goods and services. The failure to adequately respond to climate change will likely incur significant costs. However, the cost of inaction on climate change is far higher than the cost of taking action. The major global reinsurance company Swiss Re last year estimated that under a zero-mitigation scenario, up to 18% of global GDP could be lost by 2050^1^. There will be financial challenges in tackling climate change, and at Nature Communications we have curated a collection of high quality research and commentary that examines the areas of economic loss and damage, investments and technology, and economic inequality and resilience related to climate change.

One of the main financial challenges is that the economic cost of climate change will be felt unequally within and between countries. A recent study from Jun Rentschler and colleagues published in Nature Communications^2^ found that while one in five people live in high flood-risk areas, exposure risk is notably concentrated amongst lower-income households in the Global South. A growing body of research is finding that regardless of the country, climate change-attributed impacts disproportionately affect those in low income and underrepresented groups^3^. While we know that climate change mitigation has the potential to benefit future generations—particularly in lower income countries^4^, we will need equitable action at the international level as well as public-private partnerships in order to fund these efforts.

**Figure:**
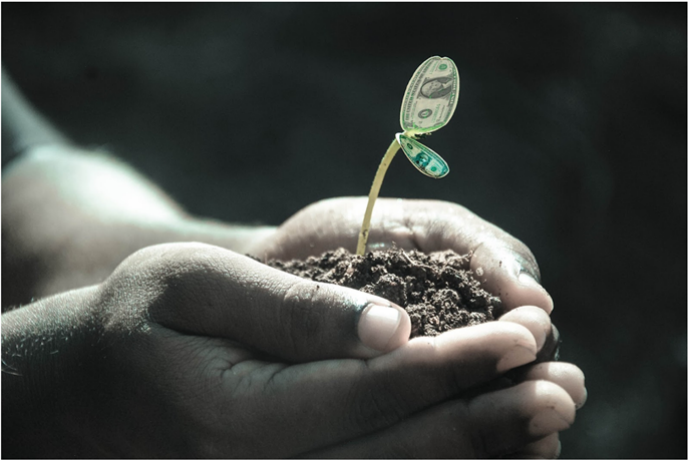
Image by Tomáš Strouhal from Pixabay

At the international level, countries have committed to addressing mitigation and supporting adaptation efforts by way of financial assistance through the Paris Agreement, and previously the Kyoto Protocol. Through these cooperative agreements, developed countries in particular, are expected to lead funding mobilization efforts, such as the UNFCCC’s Green Climate Fund. High expectations were set for discussion on loss and damage from climate change at the 2022 United Nations Climate Change Conference (COP27) in Egypt. Despite the importance of the forum, it remains to be seen whether developed countries will live up to their promises and deliver much needed financial support, as opposed to further dialogue and side stepping of responsibility, a concern raised in the countdown to COP27^5^. Lower-income countries are reliant on developed countries to commit to their promises and establish much needed funding mechanisms, and any delays in action are likely to exacerbate existing inequalities.

In public-private partnerships, climate insurance is touted as one example of cross-sector efforts to support high-risk communities, with partnerships emerging between researchers, private insurance companies, international organizations and governments. Climate insurance builds on traditional economic and insurance tools to provide financial support for those in communities at high risk from the physical impacts of climate change. It is also considered a tool to increase resilience given the additional opportunities it may provide, such as encouraging livelihood diversification due to the financial buffer. Examples of partnerships and initiatives in this area include, but not limited to, the InsuResilience Global Partnership for Climate and Disaster Risk Finance and Insurance Solutions^6^, the Munich Climate Insurance Initiative, and Oxfam America’s R4 Rural Resilience Initiative. To ensure equality of access, it is critical that insurance solutions be available to those who need it most, and affordable with respect to the most devastating of hazards. It also remains to be seen where public-private partnerships may work alongside official development assistance.

Investments are already underway in a number of critical fields—most notably in renewable energy, where there is a growing awareness that investment will help us on our pathway to decarbonization. Such investments are also understood as complementary to sustainable economic growth^7^. Although in its infancy, we are seeing increasing investment from both the public and private sectors on the research and development of emerging technologies such as carbon capture and storage to tackle CO_2_ emissions. There are certainly a number of investment challenges in these areas, not least restrictive costs for the technologies and their implementation which raises questions regarding equal access.

“We have an opportunity and a responsibility to not exacerbate inequality in an already unequal world by aiming for equitable financing solutions”

We have an opportunity and a responsibility to not exacerbate inequality in an already unequal world by aiming for equitable financing solutions. Mechanisms and agreements for climate finance require continual critical assessment, and updating as mitigation and adaptation strategies develop. Policymakers and governments – particularly in the developed world and high CO_2_ emitting countries – need to not only agree to ensuring financial support, but deliver on their promises. To not do so puts both the global economy and population at risk.
